# Volumetry as a Criterion for Suboccipital Craniectomy after Cerebellar Infarction

**DOI:** 10.3390/jcm13195689

**Published:** 2024-09-25

**Authors:** Thomas Kapapa, Andrej Pala, Burkhard Alber, Uwe Max Mauer, Andreas Harth, Hermann Neugebauer, Lisa Sailer, Kornelia Kreiser, Bernd Schmitz, Katharina Althaus

**Affiliations:** 1 Department of Neurosurgery, University Hospital Ulm, Albert-Einstein-Allee 23, 89081 Ulm, Germany; 2 Department of Neurology, Bezirkskrankenhaus Günzburg, Lindenallee 2, 89321 Ulm, Germany; 3 Department of Neurosurgery, Military Hospital Ulm, Oberer Eselsberg 40, 89081 Ulm, Germany; 4 Department of Neurology, Military Hospital Ulm, Oberer Eselsberg 40, 89081 Ulm, Germany; 5 Department of Neurology, University Hospital Ulm, Oberer Eselsberg 45, 89081 Ulm, Germany; 6 Department of Neurology, University of Wurzburg, Josef-Schneider-Strasse 11, 97080 Wurzburg, Germany; 7 Department of Paediatrics, University Hospital Ulm, Eythstrasse 24, 89075 Ulm, Germany; 8 Department of Neuroradiology, Rehabilitation Hospital Ulm, University Hospital Ulm, Oberer Eselsberg 45, 89081 Ulm, Germany; 9 Section Neuroradiology, University Hospital Ulm, Albert-Einstein-Allee 23, 89081 Ulm, Germany

**Keywords:** prognosis, stroke, outcome, ataxia

## Abstract

**Objective:** The aim of this study was to investigate the use of image-guided volumetry in cerebellar infarction during the decision-making process for surgery. Particular emphasis was placed on the ratio of the infarction volume to the cerebellar volume or cranial posterior fossa volume. **Methods:** A retrospective, multicenter, multidisciplinary study design was selected. Statistical methods such as regression analysis and ROC analysis included the volumetric data of the infarction, the posterior fossa and the cerebellum itself as new factors. **Results:** Thirty-eight patients (mean age 75 (SD: 13.93) years, 16 (42%) female patients) were included. The mean infarction volume was 37.79 (SD: 25.24) cm^3^. Patients treated surgically had a 2.05-fold larger infarction than those managed without surgery (*p* ≤ 0.001). Medical and surgical treatment revealed a significant difference in the ratio of the cranial posterior fossa volume to the infarction volume (medical 12.05, SD:9.09; surgical 5.14, SD: 5,65; *p* ≤ 0.001) and the ratio of the cerebellar volume to the infarction volume (medical 8.55, SD: 5.97; surgical 3.82, SD: 3.39; *p* ≤ 0.001). Subsequent multivariate regression analysis for surgical therapy showed significant results only for the posterior fossa volume to infarction volume ratio ≤/> 4:1 (OR: 1.162, CI: 1.007–1.341, *p* = 0.04). Younger (≤60 years) patients also had a significantly better outcome at discharge (*p* ≤ 0.017). A cut-off value for the infarction volume of 31.35 cm^3^ (sensitivity = 0.875, specificity = 0.2) was determined for the necessity of surgery. **Conclusions:** Volumetric data on the infarction, the posterior fossa and the cerebellum itself could be meaningful in decision-making towards surgery.

## 1. Introduction

Demographic change is assumed to have an impact on age-related diseases like stroke, including cerebellar infarctions, in high-income countries [1,2]. Cerebellar infarctions are more common in older adults (≥65 years) [3,4,5,6]. Furthermore, up to 54% of patients develop cerebellar edema [7,8,9,10]. Brainstem compression and occlusive hydrocephalus can result in neurologic deterioration, including coma [6,9,11]. If left untreated, this combination leads to death in about 80 to 84% of cases [10,12,13]. There is a consensus that neurosurgical measures such as the placement of external ventricular drainage and suboccipital decompression (SOD) with and without necrosectomy are indicated in the subgroup of patients with large infarcts (Class I; Level of Evidence B) [3,7,8,11,14,15,16,17,18]. Nevertheless, the benefit for the outcome after cerebellar infarction with regard to functional outcomes and independence in daily life activities is still under discussion [17,19,20,21].

SOD is recommended for patients with a cerebellar infarction who show cerebellar swelling and clinical deterioration (Glasgow Coma Score ≤ 13) over time [8,22,23]. In contrast to supratentorial decompression for infarction after the occlusion of the middle cerebral artery or traumatic brain injury, there are no randomized prospective studies and no direct recommendations for surgical decompression, particularly with regard to functional outcomes [24]. There are still too few data to support the selection of patients who would also benefit from SOD in terms of functional outcomes and to define a time point for decompression that represents a benefit for the patient [24].

Image-diagnostic volumetry of the infarcted cerebellar tissue is a well-known method of determining its size [25,26,27,28,29,30]. Unfortunately, volumetry of the cerebellar infarction is unable to better define the indication for SOD or the timing of surgery [28,31]. In other diseases, it has been shown that the relationship between the size of the cranial posterior fossa and the pathology in the posterior fossa can have prognostic relevance [32,33]. There is a need to identify predictive factors, especially for older adults, for the indication of surgical intervention and the expected outcome [3,11,30,34,35]. The aim of this study is to characterize the volumetric results of the cranial posterior fossa and the cerebral infarction that support the decision-making process for surgery or medical (non-surgical) therapy in cerebellar infarctions [7,16,30].

## 2. Materials and Methods

### 2.1. Study Design

This study represents a retrospective, multicenter, multidisciplinary study protocol. Three neurosurgery departments, three neurology departments and two neuroradiology departments took part in the study. The study conformed to the World Medical Association’s Declaration of Helsinki and was conducted after approval by the local ethics committee of the University of Ulm, Germany (application number: 86/18).

### 2.2. Inclusion and Exclusion Criteria

This study included patients from the period of 1 January 2006 to 31 December 2016, aged 18 to 99 years, who underwent imaging diagnostics via magnetic resonance imaging (MRI) or computed tomography (CT) with evidence of a cerebellar infarction during their in-hospital stay. Exclusion criteria were evidence of an acute supratentorial territorial infarction, infratentorial infarction including the brainstem or preexisting infracerebellar infarction or a pathology such as an initial intracerebral hemorrhage or traumatic brain injury. Furthermore, patients with a malignant intracranial neoplasia or a benign, space-occupying lesion of the posterior fossa were not included in the study. An infarction volume < 6 cm^3^ was also an exclusion criterion, because this would not lead to a direct indication for surgery according to the current guidelines [4,7,21,36]. In addition, patients were excluded if there was no complete documentation of the initial state of consciousness, clinical course, complete imaging (documented but not measurable images) or condition at discharge (*n* = 3).

### 2.3. Data Collection

All patients with a diagnosis of cerebellar infarction corresponding to International Statistical Classification of Diseases and Related Health Problems (ICD) code I63 were identified in the hospital’s internal database. The included patients were recorded in a separate database and various data were entered: age and sex; type of treatment; surgical procedures (insertion of external ventricular drainage (EVD), decompression of posterior fossa); pupillary function; neurological status according to the Glasgow Coma Scale (GCS) [37] (at admission and before surgical intervention or medical treatment); National Institute of Health Stroke Scale (NIHSS) [38] (at admission); Glasgow Outcome Scale (GOS) [39] at discharge; type and time of imaging diagnostics (MRI or CT); time from onset of symptoms (hours) to therapy; risk factors like weight (obesity = BMI > 24 [40]), diabetes mellitus, nicotine consumption, arterial hypertension (RR > 140/90 mmHg [41]) and coronary heart disease (CHD); and the necessity of a tracheostomy. In addition, the most common neurological symptoms presented by the patients were recorded. Then, the cerebellar infarction itself was characterized in more detail. Attention was paid to the localization, laterality and vascular territory of the infarction, and the etiology as well as the occurrence of neuroradiological mass effects like midline shift or compression of the IV ventricle. Further, we defined factors predicting unfavorable outcomes: age dichotomized ≤/>60, ≤/>75 and ≤/>80 years, weight ≥ 24 BMI, NIHSS ≥ 16, GCS ≤ 8, time to surgery/to treatment > day 3, occurrence of bilateral infarction, cerebellar midline shift ≥ 5 mm, dichotomized ratio of posterior fossa volume to infarction volume ≤/> 4:1 and dichotomized ratio of cerebellar volume to infarction volume ≤/> 3:1, infarction volume dichotomized </≥ 40 cm^3^.

### 2.4. Treatment Procedures

The patients were treated according to the international guidelines and current in-house guidelines at the time [8,42,43,44]. Within these were strategies for airway protection and oxygenation, blood pressure and glucose, temperature and prophylactic anticoagulation [43]. Patients were either admitted directly to the appropriate hospital as an emergency or transferred as a secondary transfer. The neurological condition on admission was documented using the NIHSS [38] and the GCS [37]. The primary therapy took place in a specialized stroke intensive care unit or in a stroke unit. The medical (non-surgical) therapy was carried out according to national and international standards with close monitoring of consciousness (every 2 h) and documentation using the GCS or NIHSS. If there was a deterioration in the neurological condition with a decrease in the GCS > 2 points, increase in NIHSS > 1 point or an increase in neurological deficits that could be attributed to the presence of hydrocephalus without cerebellar edema confirmed by imaging, an EVD was inserted. Hydrocephalus was defined as an age-standardized bicaudate index of >0.16 on imaging (CT or MRI) [45]. The procedure of EVD insertion was standardized in all neurosurgery departments [46]. The frontal skull was shaved and washed with antiseptic. Drill hole trepanation (3 mm and 8 mm diameter) was performed at Kocher’s point (1–2 cm in front of the coronary suture and 1.5–3 cm lateral to the midline) [47]. The dura mater was then incised, and the ventricular catheter was anatomically guided to target the lateral ventricle and advanced through the brain into the ventricle. The drill hole was closed and the catheter secured with sutures. SOD is typically performed directly after EVD. Due to the poor prognosis with progressive clinical deterioration, the indication was not influenced by previous anticoagulant or antiplatelet medication including thrombolysis. In the event of coagulation compromise, suitable reversal strategies and measures for coagulation normalization were implemented. Signs of the mass effect, such as the compression of brainstem structures, transforaminal or transtentorial herniation, compression of the fourth ventricle, midline shift or compression of the cerebrospinal fluid cisterns in the posterior fossa, were defined according to the literature [6]. The procedure of SOD was standardized in all neurosurgery departments [48]. In the prone position, the back of the head and the upper dorsal neck area were shaved and washed until sterile. A midline incision was created from the second cervical vertebra to the protuberances to provide access to the skull. The suboccipital bone was removed from the transverse sinus down to the opening of the foramen magnus in the cranio-caudal orientation and bilaterally up to the sigmoid sinus. Laminectomy of the first cervical arch, necrosectomy or a combination of both were optional and left to the surgeon’s discretion. After durotomy, a watertight duroplasty was performed. Typically, artificial foreign materials were used for the reconstruction.

### 2.5. Neuroradiological Analysis

A cerebellar infarction was diagnosed neuroradiologically by MRI or CT [49,50,51]. To exclude a brainstem infarction, the available preoperative and postoperative MRI were evaluated by experienced neuroradiologists [52,53,54]. The size of the infarction was initially calculated by experienced neuroradiologists using the ABC/2 formula [55,56]. The midline shift (MLS) was measured in millimeters (mm) before and after the respective treatment, with the midline defined as the line between the protuberantia/crista occipitalis interna and crista galli, falx dura mater or mid-clivus.

### 2.6. Outcome

The outcome was defined by the GOS at discharge because of the lack of retrospective long-term data [39]. The scale was dichotomized into unfavorable outcomes (GOS I–III) and favorable outcomes (GOS IV–V). This statistical strategy was conducted earlier in recent trials on surgical stroke therapy [47,57,58,59].

### 2.7. Volumetry

Different categories were set up to organize the neuroradiological appearances of the cerebellar infarctions. These included measuring the volume of the cerebellum, the volume of the cerebellar infarction, the volume of the posterior cranial fossa and the fourth ventricle before and after surgical or medical treatment. For statistical analysis, the image showing the greatest extent of cerebellar infarction was used, and the image that led to the indication for surgery was selected specifically for the operated patients, with respect to the limitation of CT. In the case of MRI imaging, fluid-attenuated inversion recovery (FLAIR) sequences were selected for measurement. The posterior fossa was defined as the space containing all structures caudal to the tentorium cerebelli, bordered by the occipital squama below the transverse sinuses and internal occipital protuberance (dorsal); by the dorsum sellae, the clivus, the posterior aspects of the sphenoid and the occipital bone (frontal); by the temporal bones and the lateral occipital bone (laterally); and by the parts of the parietal bones [60]. The volumetry was carried out manually using the semiautomatic “Smartbrush Tool” of the iplannet^®^ 3.0 software (BrainLab^®^, Munich, Germany), which allowed more precise infarction segmentation than the ABC/2 method. This software enables the detailed drawing of the desired structure in the respective image planes. This was performed in the axial section plane and corrected in the sagittal section plane. The software then creates a 3D image of the desired structure and calculates its volume. One measurement was conducted in collaboration with the neuroradiology department.

### 2.8. Statistical Evaluation

All characteristics of the data record to be created are first described descriptively depending on their characteristics. Qualitative variables are described using absolute and relative frequencies, while quantitatively measurable variables are described with applicable measures such as the mean, standard deviation, median and ranges (minimum and maximum values). For ordinally scaled values, the median value and the range are specified as well. In order to ensure comparability with other studies, the mean and the standard deviation are added where deemed appropriate. The authors are aware of the weaknesses of the mean and standard deviation for ordinal-scaled values. All analyses are carried out in an exploratory setting. Any comparisons of subgroups are therefore performed and interpreted, if necessary, using suitable (parametric and non-parametric) statistical tests. To perform further calculation, the dichotomized GOS at discharge (unfavorable versus favorable outcome) and therapies (surgery versus medical) were initially examined in a univariate regression analysis and then in a multivariate analysis as an explained variable to identify relevant explanatory variables from the above-mentioned factors and various variables from the database with regard to their relationships (95% confidence intervals included). Only variables that produced significant results in the univariate calculation were included in the multivariate analysis. Receiver-operating characteristic (ROC) curve and area under the curve procedures were performed to define thresholds by means of maximizing Youden’s index. The IBM SPSS Statistics Version 24 software (IBM, Armonk, NY, USA) was used for the statistical evaluation and analysis. The level of significance was set to ≤0.05.

## 3. Results

### 3.1. Patients

Thirty-eight (38) patients were identified as having therapy after a larger cerebellar infarction (>6 cm^3^). There were 22 (58%) male and 16 (42%) female patients. The mean age was 75.4 (SD:13.93). The patients could be divided into two clinical groups: (1) medical (non-surgical) therapy (*n* = 21, 55%) and (2) surgical therapy (*n* = 17, 45%). Sixteen (42%) patients underwent SOD with EVD, and one (3%) had EVD only. Three (8%) patients died during the in-hospital stay. These three patients died due to pulmonary limitations and complications, and, in one of these patients, the therapy was limited due to an existing advance directive. The majority of patients suffered from dizziness, amounting to 29 (78%); nausea and vomiting were experienced by 24 (65%) and gait disturbances by 22 (58%). In 20 (52.6%) patients, only the left cerebellar hemisphere was affected; in 11 (28.9%), the right was affected; and in seven (18.4%), both hemispheres were affected. The most frequent etiology was a cardioembolic cause in 15 (39.5%) patients, followed by arterioembolic events in seven (18.4%) patients and local thrombotic genesis in four (10.5%) patients. In 12 (31.6%) patients, no clear cause could be determined. In more than half of the patients, the vascular territory of the posterior inferior cerebellar artery (PICA) was affected, with 23 (60.5%) cases, followed by the territory of the superior cerebellar artery (SCA) with four (10.5%) patients. In six (15.8%) patients, the cause was in the vertebral artery, such as macroangiopathy. The patients’ characteristics are given in Table 1.

### 3.2. Outcome

**Figure 1:**
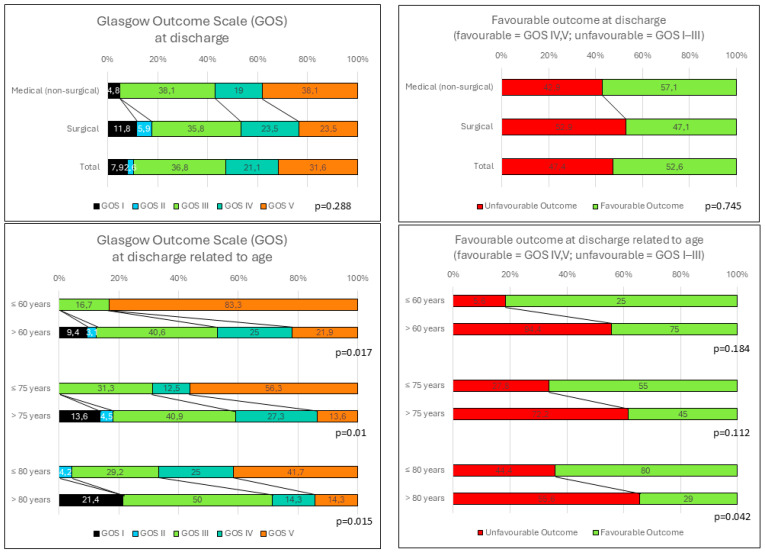
Outcomes according to the Glasgow Outcome Scale.

The distribution according to the GOS at discharge was as follows: normal state/low disability (GOS V/5) = 12 (31.6%), moderate disability (GOS IV/4) = 8 (21.1%), severe disability (GOS III/3) = 14 (36.8%), persistent vegetative state (GOS II/2) = 1 (2.6%), death (GOS I/1) = 3 (7.9%) (*p* = 0.288, Kruskal–Wallis test). The distribution according to the therapy was as follows: surgery = 8 (47.1%) favorable, medically = 12 (57.1%) favorable (*p* = 0.745, Fisher exact test) (Figure 1).

### 3.3. Volumetric Evaluation

The majority of patients had an initial CT scan (*n* = 23, 60%). MRI was used in 15 (40%) patients. CT was performed in 15 (88%) to determine the indication for SOD, and CT was also predominantly used for postoperative evaluation (*n* = 21, 55%) (Table 1). The mean cerebellar volume was 140.67 (SD: 21.69) cm^3^. The mean infarction volume was 37.79 (SD: 25.24) cm^3^ with a range of 6.3 cm^3^ to 108.6 cm^3^. The mean volume of the posterior fossa was 183.98 (SD: 27.8) cm^3^. The mean size of the 4th ventricle pre-operatively was 0.79 (SD: 0.66) cm^3^. The mean ratio of the posterior fossa volume to the infarction volume was 8.97 (SD: 8.41). The mean ratio of the cerebellar volume to the infarction volume was 6.45 (SD: 5.47) (Table 1).

In surgically treated patients the infarction volume was 52.79 (SD: 22.73) cm^3^, and, in medically treated patients, it was 25.78 (SD: 20.60) cm^3^ (*p* < 0.001, Mann–Whitney U test). Patients treated surgically had a 2.05-fold larger mean infarction volume than medically treated patients. There was not a significant difference between medically and surgically treated patients in terms of the cerebellar volume, which was 146.0 (SD:19.47) cm^3^ for surgically and 136.41 (22.90) cm^3^ for medically treated patients (*p* = 0.161, Mann–Whitney U test), or the volume of the posterior fossa, which was 183.79 (SD:26.34) cm^3^ for surgically and 184.15 (SD:29.6) cm^3^ for medically treated patients (*p* = 0.762, Mann–Whitney U test) (Table 1).

Among five (13%) patients with a midline shift of >5 mm, two (40%) patients underwent surgery and three (60%) patients had medical therapy. The mean midline shift was 2.59 (SD: 2.06) mm. There were no significant differences in the midline shift between medically (2.19 (SD: 2.19) mm) and surgically (3.27 (SD: 1.72) mm) treated patients (*p* = 0.102, Mann–Whitney U test) (Table 1).

Further, medical and surgical treatment revealed a significant difference in the ratio of the posterior fossa volume to the infarction volume (medical 12.05, SD: 9.09; surgical 5.13, SD: 5.65; *p* ≤ 0.001, Mann–Whitney U test) and the ratio of the cerebellar volume to the infarction volume (medical 8.55, SD: 5.97; surgical 3.82, SD: 3.39; *p* ≤ 0.001, Mann–Whitney U test) (Table 1). An unfavorable outcome could not be significantly associated with a specific cut-off for the infarction volume. The value of 60 cm^3^ (*p* = 0.078, Mann–Whitney U test) has the lowest error probability, but it does not meet the criterion for statistical significance.

### 3.4. Predictive Factors and the Glasgow Outcome Scale at Discharge

A univariate logistic regression analysis revealed the significant influence of the stay in the ICU in days (OR: 0.914 (CI: 0.84–0.996), *p* = 0.04), the age dichotomized into ≤80 years and >80 years (OR:0.25 (CI: 0.06–1.048, *p* = 0.05) and age (OR 0.943 (CI: 0.891–0.999), *p* = 0.05) with respect to the dichotomized GOS (Figure 1). The subsequent multivariate regression analysis showed the significance influence of the length of stay in the ICU in days (OR: 0.873 (CI: 0.776–0.981), *p* = 0.02); see Table 2.

### 3.5. Indication for Surgical Therapy

**Figure 2:**
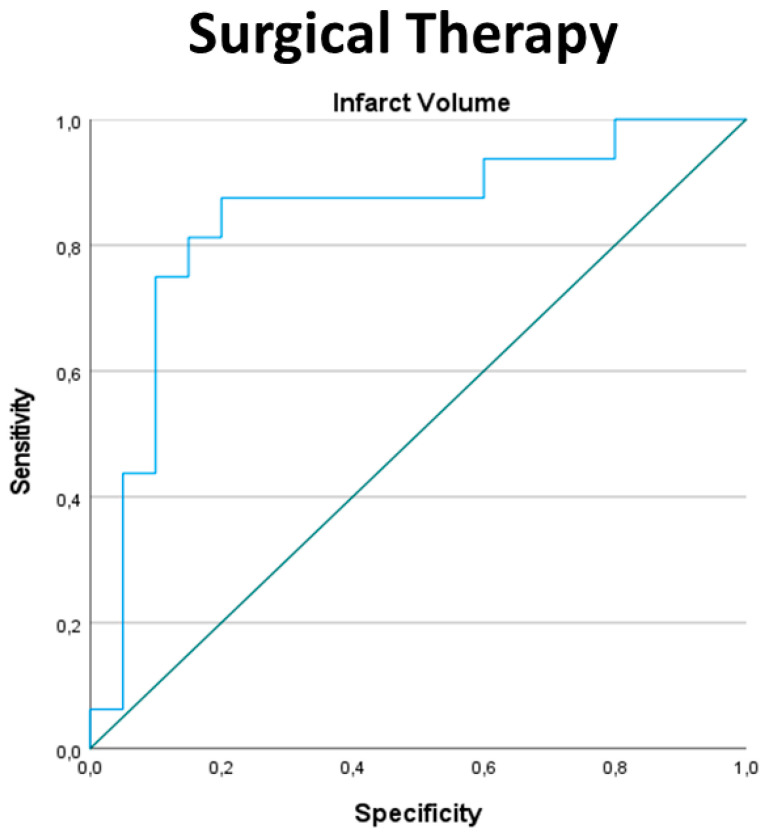
ROC analyses for the infarction volume and the decision for surgery.

A univariate analysis was performed to compare the occurrence of surgical and medical therapy. Factors with significant results were age, the GCS at admission, the infarction volume in cm^3^, the ratio of the posterior fossa volume to the infarction volume, the ratio of the cerebellar to infarction volume, the in-hospital stay in days and the duration of stay in the ICU (days) (Table 3). The subsequent multivariate regression analysis showed a significant result only for the ratio of the posterior fossa volume to the infarction volume ≤/> 4:1 (OR: 1.162 (CI: 1.007–1.341), *p* = 0.04). The ROC analysis revealed a cut-off value for the infarction volume of 31.35 cm^3^ (sensitivity = 0.875, specificity = 0.2) for surgery. A cut-off value for the ratio of the posterior fossa volume to the infarction volume of 5.21 (sensitivity = 0.9, specificity = 0.125) was calculated (Figure 2, Table 4).

### 3.6. Age-Dependent Analysis

To investigate the influence of age on the course of therapy or the outcome, different age groups were formed. According to the group classification, there were significant differences in the group sizes with regard to the dividing line ≤/> 60, 75 and 80 years (*p* < 0.001, Kolmogorov–Smirnov test) (Table 5). However, the cut-off had no significant influence on the assessment of consciousness using the GCS at admission or before the intervention, on the length of stay in the ICU or stroke unit or on the necessity of a tracheostomy, the occurrence of pupillary disorders, the infarction volume, the extent of the midline shift, the ratio of the posterior fossa volume to the infarction volume or the ratio of the cerebellar to infarction volume. A difference in the frequency of surgery was found in the comparison of patients ≤80 years and >80 years, with significantly fewer surgeries in patients > 80 years (*p* = 0.006, Mann–Whitney U test). Younger patients (≤60 years) had a significantly better outcome at discharge than those > 60 years of age (*p* ≤ 0.017, Mann–Whitney U test). A favorable outcome occurred significantly more often in patients ≤ 80 years compared to patients aged > 80 years (*p* = 0.042, Mann–Whitney U test) (Table 5).

## 4. Discussion

In a retrospective, interdisciplinary and multicenter approach, we aimed to investigate the value of diagnostic image volumetry for better decision-marking in surgery for cerebellar infarctions. In our study, the mean cerebellar infarction volume was 37.79 (SD: 25.24) cm^3^. We identified an infarction volume cut-off of 31.35 cm^3^ for surgery. In our cohort, surgical patients had a mean infarction volume that was twice that of medical patients. Univariate logistic regression analysis showed a significant influence of the infarction volume on the need for surgery, indicating that larger infarction volumes were more frequently associated with surgery. The most common procedure was SOD with the placement of an external ventricular drain. EVD as a solitary treatment was performed only in one patient, as manifested hydrocephalus is a relatively late complication of a cerebellar infarction and early SOD is necessary for brainstem decompression. Furthermore, the univariate logistic regression analysis showed that a lower ratio of the cerebellar volume to the infarction volume and a lower ratio of the posterior fossa volume to the infarction volume were associated with a higher frequency of surgery. No statistical association was found between the volumetric data and unfavorable outcomes at discharge. As a new insight, and based on our results, measuring the infarction volume and relating it to the cerebellar or posterior fossa volume may provide additional criteria for surgical decision-making.

Digital volumetry is a novel method to assess cerebellar infarctions, and, similarly to Taylor et al., we found that surgically treated patients had an average of a 41% larger infarction volume than medically treated patients [28]. The reported cerebellar infarction volume in the literature is 23 cm^3^ to 64 cm^3^ [3,7,16,55,61]. Like Taylor et al., we found that the volume of the posterior cranial fossa was not a relevant factor in our cohort [28]. Despite this fact, the ratio of the posterior fossa volume to the infarction volume was significant with regard to the need for surgery. Generally, a larger cerebellar volume and a smaller posterior fossa volume could be considered potential predictors. However, our results do not support this assumption.

Within all cerebral infarctions, the number of cerebellar infarctions is up to 3% [6,62]. With an estimated incidence of up to 54%, cerebellar edema occurs as a frequent complication in cerebellar infarctions and can lead to an acute deterioration in the patient’s condition [6,7,8,9,10]. If cerebellar edema develops, the patient’s condition can escalate to the point of coma and death [6,11,14,34,48,51,63,64]. Patients with space-occupying cerebellar infarctions can be treated medically [8]. If further clinical deterioration occurs over time, surgical measures are indicated: SOD and dural enlargement with the option of the resection of the infarcted tissue and/or ventricular drainage. Surgical therapy can significantly increase the survival rate [3,7,8,14,15,16]. However, setting the indication for surgery in older patients with comorbidities and anticoagulant medication can be particularly challenging. Long-term studies with a larger study group would be useful in order to better investigate the mortality rates and the patients’ independence in everyday life after surgery over a period of 3–5 years. Thus far, retrospective studies on this topic have only been carried out in small study groups [3,5,9,10,11,12,14,15,20,28,29,35,36,43,62,63,64,65,66,67,68]. Won et al. have recently published a larger (*n* = 531) but also retrospective cohort [21]. Factors that influence long-term life expectancy, such as the neurologic status, age, cranial nerve involvement and neuroradiologic mass effect of edema, have been found to be advantageous [20,43]. However, there is little knowledge about the clinical and neuroradiological conditions that predict the necessity of surgery and a good functional outcome after surgery [19,30]. We introduced the volumetric data of the infarction volume in relation to the cerebellar volume and the volume of the posterior fossa as a new factor supporting the indication of surgery. The benefit of these ratios of the infarction volume to cerebellar volume or the infarction volume to posterior fossa volume should be investigated prospectively and randomized.

### 4.1. Outcome after Cerebellar Infarction

The outcomes of patients with cerebellar infarctions is reported differently across studies. Calic et al. reported that, among patients with a mean age of 66 (SD: 14) years, 66% achieved a modified Rankin scale score of 0–2, 34% had a score ≥ 3, and the mortality rate was 16% after 3 months [5]. Toghi et al. described patients with a mean age of 65 (SD: 12) years, with 69% achieving a state of independence, 21% depending on help, 4% bedridden, 1% in a vegetative state and 5% dead after 3 months [62]. Macdonell et al. reported that, among patients with a mean age of 66 (45–80) years, 7% had no residual signs, 33% exhibited mild ataxia, 20% required walking aids, 17% required full nursing care and 23% had died at the time of discharge [67]. In summary, a good outcome is observed in two thirds of treated patients, with a mortality rate of less than 25% with and without surgical therapy, according to multiple studies [3,4,6,10,19,43,48]. Lindeskog et al., Pfefferkorn et al. and Juttler et al., however, reported other data, with a mean age of 53 (45–62) years, 59 (27–81) years and 60 (30–76) years, with favorable outcomes (modified Rankin Scale score of 0–3) observed in 55%, 52% and 52% of cases and unfavorable outcomes (mRS 4–6) in 45%, including 32% who died, respectively [9,12,20]. It appears that a poor outcome is more common in cohorts with a lower mean age, which suggests that volumetric data may be age-related. This should also be investigated in further studies. Nonetheless, the differences in the outcomes between these studies may be attributed to the longer observation period after the infarction. Comorbidities, the general condition and other factors, such as family circumstances and the desire for surgical treatment, are relevant aspects that may influence the decision-making in older patients, especially those over 80 years of age. These factors are certainly potential sources of bias.

Multiple factors, including cerebellar infarctions, brainstem infarctions, the secondary expansion of a cerebellar infarction, cerebellar edema, a decreased level of consciousness, hydrocephalus and hemorrhagic transformation, as well as recurrent stroke, have been associated with unfavorable outcomes [3,4,5,6,7,9,12,20,29,35,68]. Despite the demographic shift towards an increasing number of older adults with cerebellar infarctions in high-income countries, there is a lack of specific data on outcomes for this population. In our study, age and the length of stay in the ICU or stroke unit were found to be significant factors for the outcome at discharge. Our study showed significant differences in the dichotomized outcome at discharge for patients aged ≤80 years versus those >80 years, with less favorable outcomes in the latter group. Volumetry of the posterior fossa, cerebellum and infarction has the potential to provide additional factors in decision-making for SOD and EVD placement and should be considered when obtaining consent for surgery.

The time between surgical treatment and clinical deterioration is another important aspect that was not evaluated in our study. Unfortunately, due to the study design, we were unable to verify these data. In cases of cerebellar infarction, neurological deterioration is often a consequence of brainstem compression and serves as a late predictor, associated with rapid deterioration and potentially worse outcomes. Therefore, further investigation into potential anatomical or clinical predictors would be of great value.

### 4.2. Limitations of the Study

This study is retrospective. Despite the participation of four different hospitals in our study, there was only a very small number of patients that met our criteria. This limited number of cases made statistical calculations very difficult, and our results must be interpreted with caution. It should also be noted that this small number of patients again represents a selection of patients with cerebellar infarctions, as the size of the initial infarction played a role in the inclusion and exclusion criteria. Due to its nature, equivalent imaging modalities were not always used. CT might underestimate the volume of a cerebral infarction (especially in the acute setting) and has an inferior ability to differentiate vasogenic edema from and cytotoxic edema. The estimation of the cerebral infarct volume is better with MRI, especially when diffusion-weighted sequences are used. Volumetry therefore had to be performed on the different image materials available. In general, the ABC/2 method is considered to be a reliable tool in estimating clinically relevant infarct volumes. However, it has been reported several times that this method tends to underestimate the infarct volume when compared to MRI. This limitation should be acknowledged as a potential weakness of this study. This must be taken into account when interpreting the volumetry values. Furthermore, our cut-off values cannot be generalized to the broader population due to the small sample size, and the absolute numbers should not be considered representative. However, the relative size of the cerebellar infarction and posterior fossa may still assist in making therapeutic decisions in cases of equipoise. This study struggled to provide predictive factors. The retrospective approach made the interpretation of the given clinical factors challenging. We were not able to obtain long-term outcome data for all patients. In about one third of the patients, the 12-month outcome could not be determined. Hence, we calculated the outcome on the basis of the outcome at discharge. The isolated larger cerebellar infarction seems to be a rare clinical finding. The conclusions of this study cannot be extended to patients with brainstem infarctions and/or hemorrhagic transformed strokes. In order to improve cerebellar infarction studies, a prospective concept should be developed. A randomizing concept that distinguishes between surgical and non-surgical forms of therapy would also be desirable. Due to the high mortality of non-surgical therapy, however, this scientific approach is difficult to implement [9]. Therefore, future investigations should focus on the prediction of surgical interventions and the factors predicting surgical interventions in an aging population.

## 5. Conclusions

Volumetry of the posterior fossa, cerebellum and infarction may help in decision-making for SOD and EVD placement and should be considered when obtaining consent for surgery. However, further studies are needed to confirm this.

## Figures and Tables

**Table 1:** Patients’ characteristics (National Institute of Health Stroke Scale = NIHSS, Glasgow Coma Score = GCS).

*n* = 38	Medical (*n* = 21, 55%)	Surgery (*n* = 17, 45%)	Total (*n* = 38, 100%)	*p*
**Age** (years, SD)	80.1 (12.94)	69.5 (13.15)	75.4 (13.93)	**0.017**
**Gender**				
Female	9 (43)	7 (41)	16 (42)	1.000
**Imaging on admission**				
Magnet Resonance Imaging	10 (48)	5 (29)	15 (40)	0.326
Computed Tomography	11 (52)	12 (71)	23 (60)	
**Imaging pre-surgery**				
Magnet Resonance Imaging		2 (12)		
Computed Tomography		15 (88)		
**Imaging for follow-up/post-surgery**				
Magnet Resonance Imaging	11 (52)	6 (35)	17 (45)	0.342
Computed Tomography	10 (48)	11 (65)	21 (55)	
**Clinical symptoms *n* (%)**				
Cephalgia	6 (30)	12 (70)	18 (49)	**0.022**
Disturbance of consciousness	4 (19)	11 (65)	15 (40)	**0.007**
Dizziness	16 (80)	13 (77)	29 (78)	1.000
Nausea/vomiting	14 (70)	10 (59)	24 (65)	0.512
Facial paresis	3 (15)	1 (6)	4 (11)	0.609
Speech disorders	10 (48)	8 (47)	18 (47)	1.000
Gait disturbances	13 (62)	9 (53)	22 (58)	0.743
Ocular motility disorder	8 (40)	8 (47)	16 (43)	0.746
Disturbances of coordination	15 (75)	7 (44)	22 (61)	0.087
Initial NIHSS on admission, Mean (SD)	4.4 (6.89)	5.7 (6.56)	5 (6.65)	0.398
Median (Range)	2 (0–25)	3 (0–18)	2 (0–25)	
GCS pre-interventional, Mean (SD)	13.3 (3.39)	10.5 (4.83)	12.1 (4.26)	**0.006**
Median (Range)	15 (4–15)	14 (3–15)	15 (3–15)	
GCS on admission, Mean (SD)	14.2 (2.68)	14.4 (1.97)	14.3 (2.36)	0.532
Median (Range)	15 (4–15)	15 (7–15)	15 (4–15)	
Anisocoria (%)	4 (19)	1 (6)	5 (13)	0.355
ICU stay in days, Mean (SD)	4.8 (8.35)	15.1 (13.1)	9.2 (11.7)	**0.002**
Artificial ventilation in hours, Mean (SD)	43.1 (102.0)	48.5 (67.77)	45.5 (87.31)	**0.003**
**Risk factors *n* (%)**				
Diabetes mellitus	3 (14)	7 (41)	10 (26)	0.078
Nicotine abuse	3 (14)	6 (35)	9 (24)	0.249
Arterial hypertension	17 (81)	16 (94)	33 (87)	0.355
History of vascular events	6 (29)	7 (41)	13 (34)	0.502
Coronary heart disease	0 (0)	6 (35)	6 (16)	**0.004**
Obesity	5 (24)	8 (47)	13 (34)	0.178
**Duration between symptom onset and surgery in hours, Mean (SD)**		48.53 (32.51)		
**Vascular territory *n* (%)**				
Vertebral artery	4 (19)	2 (12)	6 (16)	0.801
Posterior inferior cerebellar artery	11 (52)	12 (71)	23 (61)	
Anterior inferior cerebellar artery	2 (10)	1 (6)	3 (8)	
Superior cerebellar artery	3 (14)	1 (6)	4 (11)	
Unknown	1 (5)	1 (6)	2 (5)	
**GOS at discharge**				
Mean (SD)	3.9 (1.11)	3.4 (1.28)	3.7 (1.19)	0.288
Median (Range)	4 (1–5)	3 (1–5)	4 (1–5)	
**In-hospital outcome *n* (%)**				
Death	1 (5)	2 (12)	3 (8)	0.577
**Volumetric data**				
Cerebellar volume (cm^3^) (SD)	136.41 (22.90)	146.0 (19.47)	140.67 (21.69)	0.161
Volume cerebellar infarction (cm^3^) (SD)	25.78 (20.60)	52.79 (22.73)	37.79 (25.24)	**<0.001**
Volume cranial posterior fossa (cm^3^) (SD)	184.14 (29.59)	183.79 (26.33)	183.98 (27.80)	0.762
Volume IV ventricle (pre-surgery) (cm^3^) (SD)	0.79 (0.64)	0.80 (0.71)	0.79 (0.66)	0.883
Volume IV ventricle (post-surgery) (cm^3^) (SD)	0.96 (0.66)	0.35 (0.19)	0.66 (0.57)	**0.001**
Midline shift (pre-surgery) (mm) (SD)	2.19 (2.19)	3,27 (1.72)	2.59 (2.06)	0.102
Ratio posterior fossa: infarct volume (SD)	12.05 (9.09)	5.13 (5.65)	8.97 (8.41)	**<0.001**
Ratio cerebellar: infarct volume (SD)	8.55 (5.97)	3.82 (3.39)	6.45 (5.47)	**<0.001**

**Table 2:** Univariate and multivariate regression analysis for unfavorable outcome (Glasgow Outcome Scale I–III, ICU = intensive care unit).

Univariate				
Factor	Odds Ratio	95% Confidence Interval		*p*
		Lower	Upper	
Stay in ICU (days)	0.914	0.84	0.996	0.04
Age > 80 years	0.250	0.06	1.048	0.05
Age	0.943	0.891	0.999	0.05
**Multivariate**				
**Factor**	**Odds Ratio**	**95% Confidence Interval**		***p***
		**Lower**	**Upper**	
Stay in ICU (days)	0.873	0.776	0.981	0.02

**Table 3:** Univariate and multivariate regression analysis for surgical therapy (ICU = intensive care unit).

Univariate				
Factor	Odds Ratio	95% Confidence Interval		*p*
		Lower	Upper	
Age	1.067	1.006	1.131	0.03
GCS at admission	1.184	0.997	1.406	0.05
Infarct volume	0.942	0.904	0.982	0.005
Ratio of posterior fossa volume to infarct volume	1.171	1.007	1.361	0.04
Ratio of cerebellar volume to infarct volume	1.306	1.027	1.661	0.029
In-hospital stay in days	1.166	1.018	1.336	0.027
Stay in ICU (days)	0.888	0.808	0.975	0.013
Infarct volume ≥ 40 cm^3^	0.058	0.011	0.305	<0.001
Infarct volume ≥ 50 cm^3^	0.067	0.011	0.394	0.003
Infarct volume ≥ 55 cm^3^	0.111	0.019	0.645	0.014
Infarct volume > 59 cm^3^	0.068	0.007	0.636	0.018
**Multivariate**				
**Factor**	**Odds Ratio**	**95% Confidence Interval**		***p***
		**Lower**	**Upper**	
Ratio of posterior fossa volume to infarct volume > 4:1	1.162	1.007	1.341	0.04

**Table 4:** Sensitivity and specificity values for the threshold variables.

ROC Curve Analysis for All Patients, Surgical Therapy	Infarction Volume cm^3^	Ratio of Posterior Fossa Volume to Infarction Volume	Ratio of Cerebellar to Infarction Volume
AUC	0.841	0.841	0.838
Standard error	0.73	0.076	0.076
*p*	0.001	0.001	0.001
CI 95%: lower	0.698	0.692	0.688
CI 95%: upper	0.983	0.989	0.987
Cut-off	31.35	5.2127	3.7957
Sensitivity	0.875	0.900	0.900
Specificity	0.200	0.125	0.125

**Table 5:** Impact of age on different variables (ICU = intensive care unit, MLS = midline shift).

	Age Classification (Years)								
	≤60	>60	*p*	≤75	>75	*p*	≤80	>80	*p*
***n*** **(%)**	6 (10.7)	32 (89.3)	**<0.001**	16 (35.2)	22 (64.8)	**<0.001**	24 (56.8)	14 (43.2)	**<0.001**
**Age**									
**Mean (SD)**	51.0 (6.92)	79.9 (9.32)	**<0.001**	63.1 (11.07)	84.4 (7.35)	**<0.001**	67.8 (11.3)	88.4 (6.28)	**<0.001**
**Median (range)**	54 (40–58)	78 (61–96)		66 (40–75)	82 (76–96)		73 (40–79)	87.5 (80–96)	
**Glasgow Coma Score**									
**Mean (SD)**	10.5 (5.86)	12.4 (3.93)	0.225	11.94 (4.85)	12.2 (3.87)	0.728	12.1 (4.46)	12.1 (4.03)	0.891
**Median (range)**	13.5 (3–15)	15 (3–15)		14.5 (3–15)	15 (3–15)		15 (3–15)	15 (3–15)	
**Surgery *n* (%)**	4 (66.7)	13 (40.6)	0.378	10 (62.5)	7 (31.8)	0.099	15 (62.5)	2 (14.3)	**0.006**
**Medical *n* (%)**	2 (33.3)	19 (59.4)		6 (37.5)	15 (68.2)		9 (37.5)	12 (85.7)	
**Stay in ICU: Mean (SD)**	7.17 (6.08)	9.65 (12.53)	0.966	11.63 (14.76)	7.43 (8.67)	0.471	10.22 (12.94)	7.64 (9.56)	0.564
**Tracheostoma (*n* =** **5, 13.16%): *n*(%)**	0 (0)	5 (100)	0.401	2 (40)	3 (60)	0.654	4 (80)	1 (20)	0.381
**Anisocoria (*n* =** **5, 13.16%): *n*(%)**	1 (20)	4 (80)	0.599	1 (20)	4 (80)	0.286	3 (60)	2 (40)	0.619
**Infarct volume in cm^3^, Mean (SD)**	39.6 (19.27)	37.49 (26.33)	0.599	45.30 (22.91)	33.0 (25.98)	0.077	42.77 (28.09)	29.97 (18.27)	0.178
**MLS in mm, Mean (SD)**	2.46 (1.76)	2.61 (2.13)	1	3.12 (2.18)	2.24 (1.95)	0.242	2.64 (2.12)	2.54 (2.07)	0.864
**Ratio of posterior fossa volume to infarct volume, Mean (SD)**	7.97 (7.98)	9.14 (8.59)	0.945	7.49 (9.03)	9.90 (8.05)	0.136	8.88 (9.13)	9.13 (7.46)	0.284
**Ratio of cerebellar to infarct volume, Mean (SD)**	5.40 (4.30)	6.61 (5.67)	0.982	5.37 (5.90)	7.16 (5.19)	0.144	6.33 (5.96)	6.64 (4.79)	0.284
**Glasgow Outcome Scale**									
**Mean (SD)**	4.67 (0.82)	3.47 (1.16)	**0.017**	4.25 (0.931)	3.23 (1.19)	**0.01**	4.04 (0.96)	3 (1.3)	**0.015**
**Median (range)**	5 (3–5)	4 (1–5)		5 (3–5)	3 (1–5)		4 (2–5)	3 (1–5)	
**Unfavorable outcome *n* (%)**	1 (16.7)	17 (53.1)	0.184	5 (31.3)	13 (59.1)	0.112	8 (33.3)	10 (71.4)	**0.042**
**Favorable outcome *n* (%)**	5 (83.3)	15 (46.9)		11 (68.8)	9 (40.9)		16 (66.7)	4 (28.6)	

## Data Availability

Data are given in the manuscript. Raw data are available from the corresponding author by request.
